# Norovirus (NoV) specific protective immune responses induced by recombinant P dimer vaccine are enhanced by the mucosal adjuvant FlaB

**DOI:** 10.1186/s12967-016-0899-4

**Published:** 2016-05-17

**Authors:** Vivek Verma, Wenzhi Tan, Sao Puth, Kyoung-Oh Cho, Shee Eun Lee, Joon Haeng Rhee

**Affiliations:** Clinical Vaccine R&D Center, Chonnam National University Medical School, Gwangju, South Korea; Department of Pharmacology and Dental Therapeutics, School of Dentistry, Chonnam National University, Gwangju, South Korea; Department of Microbiology, Chonnam National University Medical School, Gwangju, South Korea; Laboratory of Veterinary Pathology, College of Veterinary Medicine, Chonnam National University, Gwangju, South Korea; Georgia Cancer Center, Augusta University, 1410 Laney Walker Blvd, Augusta, 30912 Georgia USA

**Keywords:** Norovirus, FlaB, Adjuvant, Antibody, Intranasal, Sublingual, Subcutaneous, Alum

## Abstract

**Background:**

Noroviruses (NoVs) are a major cause of childhood gastroenteritis and foodborne diseases worldwide. Lack of appropriate animal models or cell-based culture systems makes the development and evaluation of NoV-specific vaccines a daunting task. VP1 is the major capsid protein of the NoVs that acts as a binding motif to human histo-blood group antigens (HBGAs) through its protruding 2 (P2) domain and can serve as a protective antigen candidate for vaccine development.

**Methods:**

Recombinantly produced NoV specific P domain (Pd) vaccine was inoculated into groups of mice either alone or in conjugation with mucosal adjuvant FlaB, the flagellar protein from *Vibrio vulnificus*. Antigen specific humoral and cell mediated immune responses were assessed by enzyme linked immunosorbent assay (ELISA) or fluorescent activated cell sorting (FACS). A comparative analysis of various routes of vaccination viz. intranasal, sublingual and subcutaneous, was also done.

**Results:**

In this study, we show that a recombinant Pd-vaccine administered through intranasal route induced a robust T_H_2-dependent humoral immune response and that the combination of vaccine with FlaB significantly enhanced the antibody response. Interestingly, FlaB induced a mixed T_H_1/T_H_2 type of immune response with a significant induction of IgG1 as well as IgG2a antibodies. FlaB also induced strong IgA responses in serum and feces. FlaB mediated antibody responses were toll like receptor 5 (TLR5) dependent, since the FlaB adjuvanticity was lost in TLR5^−/−^ mice. Further, though the Pd-vaccine by itself failed to induce a cell mediated immune response, the Pd-FlaB combination stimulated a robust CD4^+^IFNγ^+^ and CD8^+^IFNγ^+^ T cell response in spleen and mesenteric lymph nodes. We also compared the adjuvant effects of FlaB with that of alum and complete Freund’s adjuvant (CFA). We found that subcutaneously inoculated FlaB induced more significant levels of IgG and IgA in both serum and feces compared to alum or CFA in respective samples.

**Conclusion:**

We validate the use of TLR5 agonist as a strong mucosal adjuvant that would facilitate the development of NoV specific vaccines for humans and veterinary use. This study also highlights the importance of route of immunization in inducing the appropriate immune responses in mucosal compartments.

## Background

Human noroviruses (NoVs) are the leading cause of childhood gastroenteritis and foodborne diseases worldwide [1]. Human NoVs, belonging to the genus Norovirus within the family Caliciviridae, are a group of small, positive sense, single-stranded, non-enveloped viruses having 7–8 kb RNA genome with four open reading frames (ORFs) [2, 3]. Major and minor capsid proteins VP1 and VP2 are encoded by the ORF2 and 3, respectively [4]. ORF1 encodes the nonstructural protein and ORF4 has been described recently to play a significant role in the murine NoV pathogenesis [3, 5]. The absence of efficient and reproducible cell culture systems and small-animal models has hindered the studies concerning the pathogenesis and molecular mechanisms of NoV life cycle as well as the development of effective vaccines and therapeutic agents [5, 6].

**Fig. 1:**
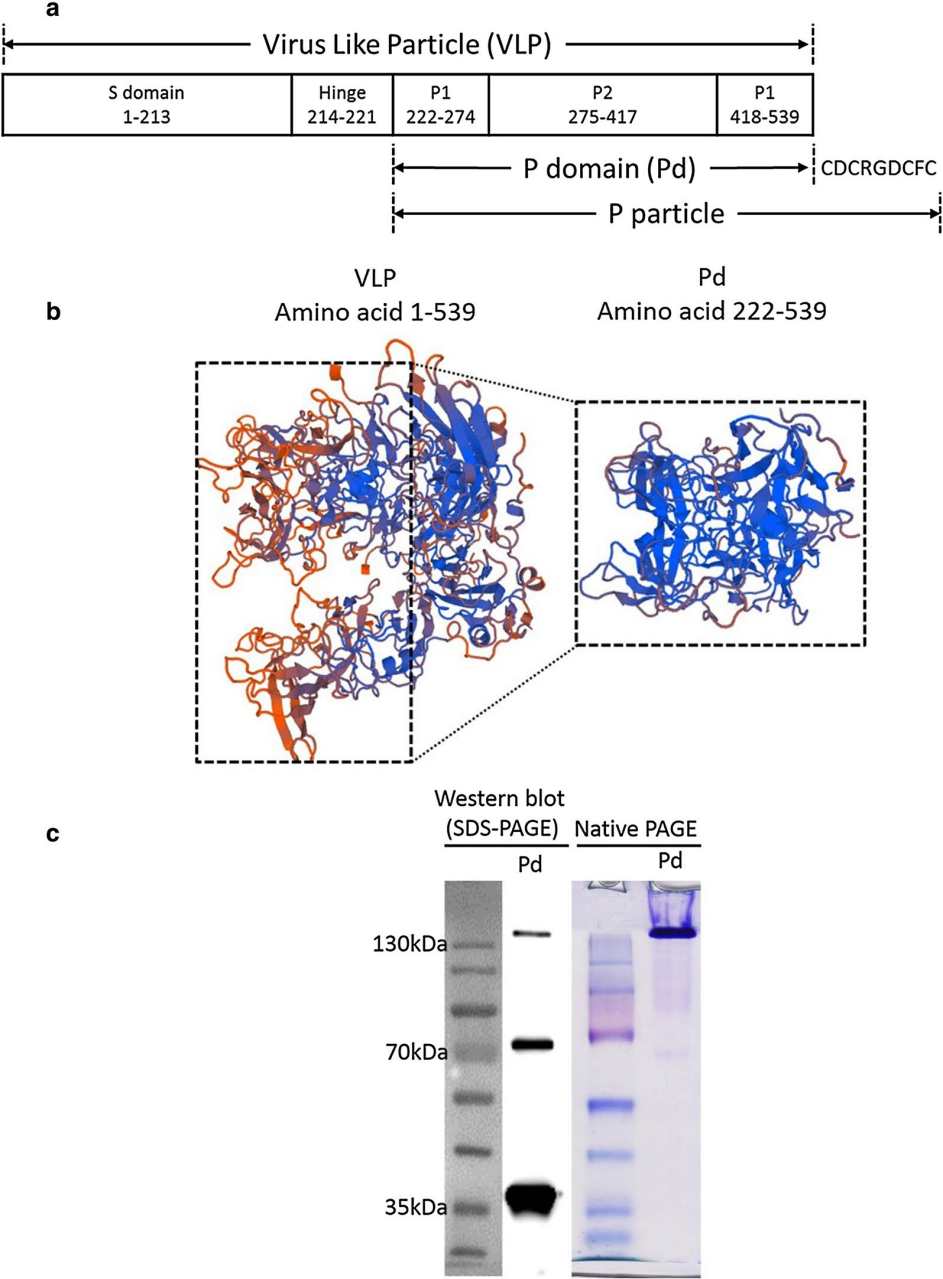
Antigen construction and expression. **a** Nomenclature and location of various domains of NoV VLPs. **b** 3D structure of VP1 encoded by ORF2 of human NoV (GII.4) (GenBank accession# AY038600.3) and of the P domain (Pd) cloned into bacterial vector system (for details see ‘‘methods’’). Numbers in (**a**, **b**) represent the amino acid numbers. **c** The Western blot of the bacterially expressed and purified Pd protein reacted with anti-Pd hyper-immune serum raised in mice. The recombinant Pd protein was separated by the native PAGE

Virus-like particle (VLP)-vaccines have been developed and approved for the prevention of hepatitis B virus (HBV) [7, 8] and human papillomavirus (HPV) [9, 10] infections. The capsid proteins of NoVs have been characterized to generate VLPs (comprised of major structural protein VP1), P particle (comprised of P domain with –C terminal CDCRGDCFC peptide) and P domain (Pd also called P dimer) (Fig. 1a) [11–13]. The structural variants of NoV VLPs have been shown to be presented efficiently by dendritic cells (DCs) and elicit efficient serum IgG antibody responses [11]. Given NoVs are pathogens of gastrointestinal tract that cause pathology primarily in the small intestine, effective vaccines should induce significant secretory antibodies in gastrointestinal tract to effectively control viral invasion and shedding in feces, and restrict the human-to-human spread. Subunit protein vaccines (e.g. Pd-vaccine) are poor inducers of humoral immune responses, in particular secretory antibody responses [14]. In this context, the use of adjuvants potentiating immune responses in the mucosal immune compartments will significantly boost the vaccine efficacy [15]. One particular group of immune-enhancers that can be exploited as mucosal adjuvants is toll like receptors (TLRs) [16]. TLRs are pattern recognition receptors (PRRs) that specifically recognize their cognate agonist(s), also known as pathogen associated molecular patterns (PAMPs). The TLR agonists include bacterial products, CpG motifs, virus-specific nucleotides (i.e. dsRNA) and imidazoquinoline compounds [17]. In our previous studies we have shown that FlaB, a flagellin protein from *Vibrio vulnificus* and an agonist of TLR5, is a potent adjuvant for mucosally administered vaccines [18]. In the present study, we evaluated the potential of FlaB coadministration along with the recombinant Pd antigen in enhancing the antigen-specific protective immune responses. We report that the intranasal immunization of mice with Pd+FlaB mixture vaccine induced a potent antibody and cell mediated immune response in both systemic and mucosal compartments. We also compared the immune efficacy after vaccine administration through sublingual route. Additionally, we evaluated the advantages of FlaB over known vaccine adjuvants such as alum and complete Freund’s adjuvant (CFA).

## Methods

### Antigen preparation and LPS removal

Norovirus VA387 strain (GII.4) P dimer-specific DNA fragments were cloned between *Nde*I-*Kpn*I restriction sites and expressed in pET30a+ vector (Novagen) as a His-tagged protein. All proteins were expressed in *Escherichia coli* BL21 with an induction by 0.1 mM isopropyl-β-d-thiogalactopyranoside (IPTG) at 37 °C overnight. Bacterial pellets were dissolved in 8 M urea followed by sonication (2 s on to 3 s off cycles/5 min at 30 % of max. voltage) on ice. Following centrifugation (8000 rpm/15 min/4 °C), protein from cell free supernatant was purified by affinity purification using Ni–NTA Agarose beads (Qiagen) as per manufacturer’s instructions. Protein was dialyzed extensively against sterile phosphate buffered saline (PBS) followed by LPS removal by treatment with TritonX-114 (Sigma). Traces of Triton X-114 were removed by treatment with Bio-Beads™ SM-2 (Bio Rad) as per manufacturer’s instructions. For the production of FlaB protein, a 1.5-kb fragment containing the open reading frame of *V. vulnificusflaB* was cloned into pTYB12-yielding pCMM250 (New England Biolabs). Recombinant FlaB was purified as previously reported [18]. Finally all proteins were suspended in sterile PBS at appropriate concentrations.

### Animals, vaccination and sampling

Specific pathogen free (SPF) female Balb/c WT mice were purchased from Charles River Inc. while TLR5^−/−^ mice on Balb/c background were bred and maintained under SPF conditions at the animal facility of Clinical Vaccine R&D Center of Chonnam National University. The mouse study protocol was approved by the Committee on Animal Welfare at Chonnam National University Medical School. Mice were immunized at an age of 6–7 weeks. The animals were housed in a temperature- and light-controlled environment and had free access to food and water. Various antigen combinations were used at equimolar concentrations. Vaccination groups included (1) P dimer (Pd), (2) Pd + FlaB, (3) FlaB, and (4) PBS. All antigens were inoculated through intranasal (i.n.) or sublingual (s.l.) routes into anaesthetized animals. Final volume for i.n. as well as s.l. vaccination was 10 μl/animal. In a separate experiment, groups of five mice were immunized subcutaneously with either alum precipitated Pd [19], Pd-CFA mixture, or Pd+FlaB mixture. In all the adjuvant groups, concentration of Pd antigen inoculated into mice was kept constant at 0.1 μM/dose. Animals were immunized thrice. In the CFA group, the first vaccination was done with CFA + Pd followed by two immunizations at one-week interval with incomplete Freund’s adjuvant along with Pd. In all immunization groups, before each respective vaccination, mouse serum as well as feces were collected and processed for antibody determination. One week after the third immunization, final blood and feces samples from mice were procured. Feces samples were made into a 20 % solution (w/v) in ice cold PBS containing 1 mM phenylmethylsulfonyl fluoride (PMSF). All clarified serum and feces samples were kept at −80 °C until used.

### NoV specific enzyme linked immunosorbent assay (ELISA)

Antibody titers in serum and feces samples from individual animals were estimated by ELISA using Pd as the coating antigen. Samples were serially diluted in a dilution buffer (PBS + 1 % BSA + 0.05 %Tween20). Anti-mouse IgG, IgA, IgG1 and IgG2a specific Horse radish peroxidase (HRP) conjugated secondary antibodies (Southern Biotech) were used at a dilution of 1:2000. Finally HRP-specific optical reactions were developed using the BD OptEIA™ (BD Biosciences, San Diego, USA) for 15 min followed by stopping the reaction with 2 N H_2_SO_4_. Reactions were read at OD_450_ using Spectra Max 190 microplate reader (Molecular Devices). The cut-off value was decided as per formula: (Mean OD_450_ of negative control wells) + 3 (standard deviation of OD_450_ of negative control wells). Log_2_ of the reciprocal of highest dilution showing OD_450_ value equivalent to or higher than the cut off value was taken as the antibody titer.

### Tissue processing, lymphocyte isolation, cell stimulation assay and flow cytometry

Spleen, mesenteric lymph nodes (MLNs) and Payer’s patches (PPs) were harvested from animals in various immunization groups 2 weeks after the final immunization and kept in ice-cold cell culture medium until processed. All tissues were mashed through 40 μm cell strainer (BD Falcon). In case of spleen, lymphocytes were purified by density centrifugation using the Lymphoprep™ (Life Technologies) followed by red cells lysis by ACK lysis buffer (Lonza Inc., USA) for 5 min at room temperature (RT). Cells were finally suspended in the cell culture medium (RPMI1640 + 10 % FBS + 1 % Penicillin/Streptomycin) and kept on ice until used. For in vitro stimulation assays, 1 × 10^6^ cells in duplicate were plated in separate flat-bottom 96 wells and stimulated with 1 μg of Pd protein for 12 h. Golgistop™ (BD Biosciences, San Diego, CA) was added for the entire duration of incubation. Post-incubation cells were fixed and permeabilized using the Cytofix/Cytoperm kit (BD Biosciences) in accordance with the manufacturer’s instructions. The anti-mouse antibodies used for intracellular and surface staining (eBioscience or BD Pharmingen™) were CD4-APC (clone GK1.5), CD8-APC (clone 53–6.7) and IFNγ-PE (clone XMG1.2). Stained cells were acquired on FACS Accuri (BD Biosciences). Data analysis was performed using the FlowJo software (TreeStar, San Carlos, CA).

### Statistical analysis

Statistical analysis of data was done using the GraphPad Prism software ver. 6.0 or Microsoft Excel as appropriate. P < 0.05 was taken as significant.

## Results

### Cloning and expression of NoV P dimer (Pd)

Norovirus specific P dimer (Pd) containing two P1 domains and one central P2 domain (Fig. 1a) was expressed as a recombinant protein in *Escherichia coli* BL21. Homology modelling [20] suggested that though the complete viral protein had slightly dispersed structure (Fig. 1b), the P domain from ORF2 encompassing amino acid 222–539 had globular structure that would tend to have a compact three dimensional (3D) organization (Fig. 1b). This observation was substantiated by the non-denaturing native polyacrylamide gel electrophoresis (PAGE) of the cloned protein that showed that most of Pd protein remained in the stacking gel (Fig. 1c). The Western blot analysis from sodium dodecyl sulphate (SDS)-PAGE showed that the recombinantly produced protein had a major single monomeric unit of 37 kDa while minor fractions of ca. 80 kDa and over 120 kDa, representing dimer and multimeric proteins respectively, were also present (Fig. 1b).

### FlaB enhances Pd-specific antibody responses in a TLR5-dependent manner

**Fig. 2:**
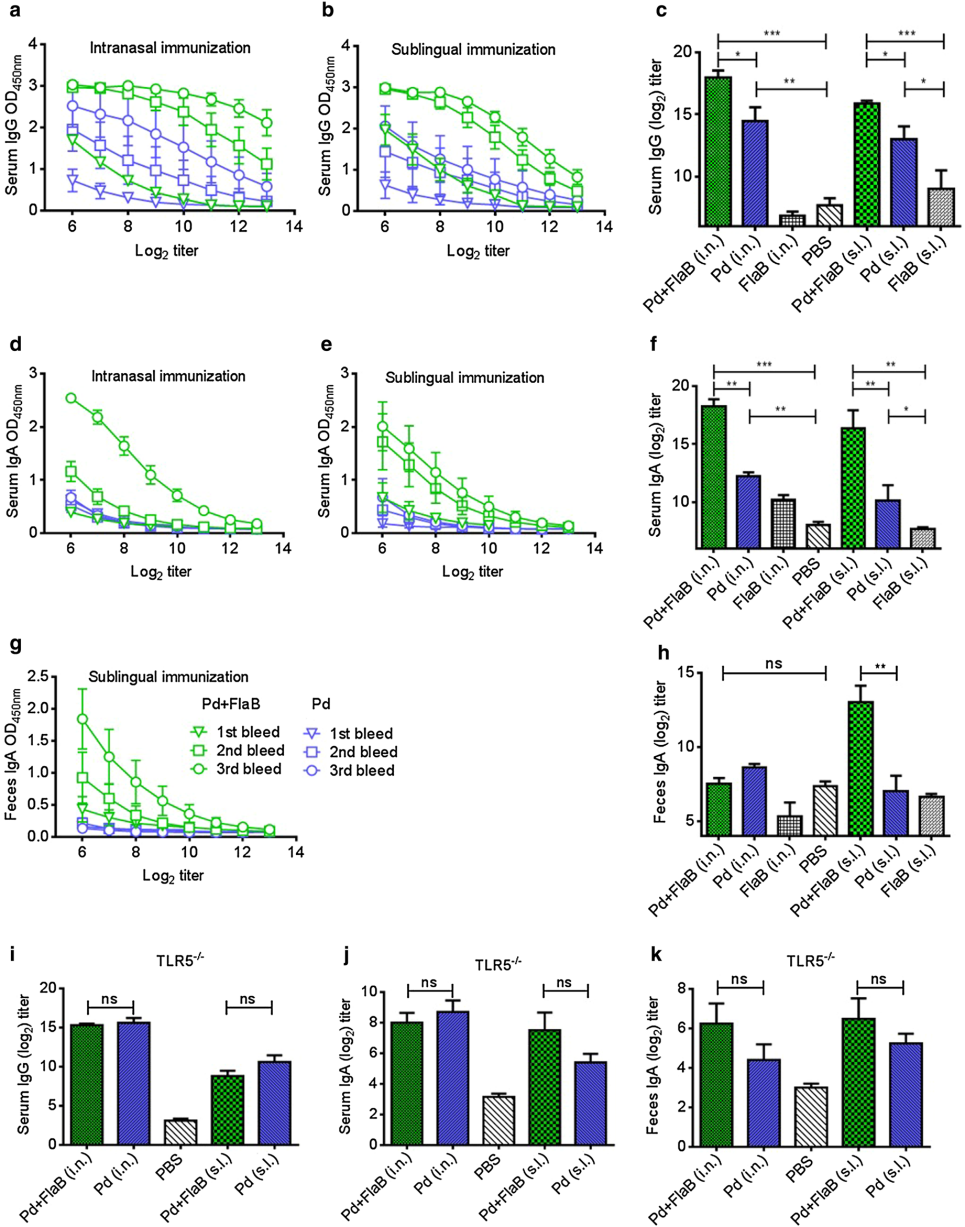
Induction of humoral immune response post-immunization. **a**, **b** The kinetics of appearance of IgG after immunization with Pd or Pd + FlaB given through intranasal (i.n.) and sublingual (s.l.) routes respectively. **c** The comparative serum-IgG antibody titers after final vaccination. **d**, **e** The kinetics of appearance of serum IgA antibodies after i.n. and s.l. inoculations of various antigens. The legends for (**a**), (**b**), (**d**), (**e**) are in (**g**). **f** The serum-IgA levels 1 week after final immunization. **g** The kinetics of appearance of feces-IgA after s.l. immunization. (**h**) The final feces-IgA titers 1 week after final vaccination. **i**–**k** The respective serum IgG, serum IgA and fecal IgA antibody titers from TLR5 ^−/−^ mice. Statistical differences were compared by the Student’s *t* test for unpaired means. *P < 0.05, **P < 0.01, ***P < 0.001, ^ns^not significant. The *error bars* represent the standard error of mean (SEM)

In order to estimate the immunogenicity of P dimers expressed in bacterial system, mice were immunized through i.n. or s.l. route using Pd antigen alone or in combination with FlaB (Pd + FlaB). FlaB and PBS were included as negative controls. For the estimation of the kinetics of anti-Pd specific immune responses, serum and feces samples, taken before each vaccination and 1 week after the final vaccination, were assayed by ELISA. After vaccination with Pd, we noted a steady increase in serum IgG levels that peaked only after third immunization. On the other hand, the immunization with Pd + FlaB induced a strong immune response even after the first immunization that resulted in significantly high serum IgG levels (Fig. 2a, b). Nonetheless, the antibody responses appeared to be boosted significantly by repeated immunizations even in the Pd only immunized groups. Particularly, immunization with Pd resulted in significantly higher IgG responses in serum of immunized animals through both i.n. (14.5 ± 2.16, log_2_ titer ± SD; P = 0.002) as well as s.l. routes (13 ± 2.08; P = 0.003) (Fig. 2c). However, presence of FlaB along with Pd (Pd + FlaB) very significantly enhanced the antigen-specific (18 ± 1.08; P < 0.0001) immune responses when given through i.n. route (Fig. 2c). On the other hand, the immune responses induced after s.l. immunization using Pd + FlaB (15.875 ± 0.478) were also significantly higher than the negative control groups (P < 0.05) but were significantly less than the respective immunization done through i.n. route (Fig. 2a–c). The IgA antibody responses in the serum showed similar patterns with those of IgG antibodies (Fig. 2d and f). The i.n. immunization using Pd + FlaB induced the most potent serum-IgA response (18.25 ± 1.258) that was significantly higher than that after immunization with Pd alone (12.25 ± 0.645; P = 0.0005) (Fig. 2d, f). The s.l. immunization route manifested similar results (Fig. 2e) though the titers were slightly lower than the i.n route (Fig. 2d). However, highest IgA levels in feces were induced upon vaccination with Pd + FlaB through s.l. route (13 ± 2.273) (Fig. 2g) that was significantly higher than any other groups irrespective of immunization routes (Fig. 2h). No significant IgG levels were detected in the feces in any immunization groups (data not shown). As expected, the enhancement of Pd specific antibody titers by FlaB was mediated through TLR5 since significant increases in antigen-specific antibody titers were not observed in TLR5^−/−^ mice (Fig. 2i–k). Though the levels of fecal-IgA in TLR5^−/−^ mice induced upon Pd + FlaB immunization tended to be slightly higher than Pd alone, but the difference did not reach a statistical difference (Fig. 2k). These data indicate that FlaB significantly induced mucosal and systemic IgA and IgG responses in TLR5 dependent manner.

### FlaB polarizes a mixed T_H_1/T_H_2 type humoral immune response against NoV Pd-vaccine

**Fig. 3:**
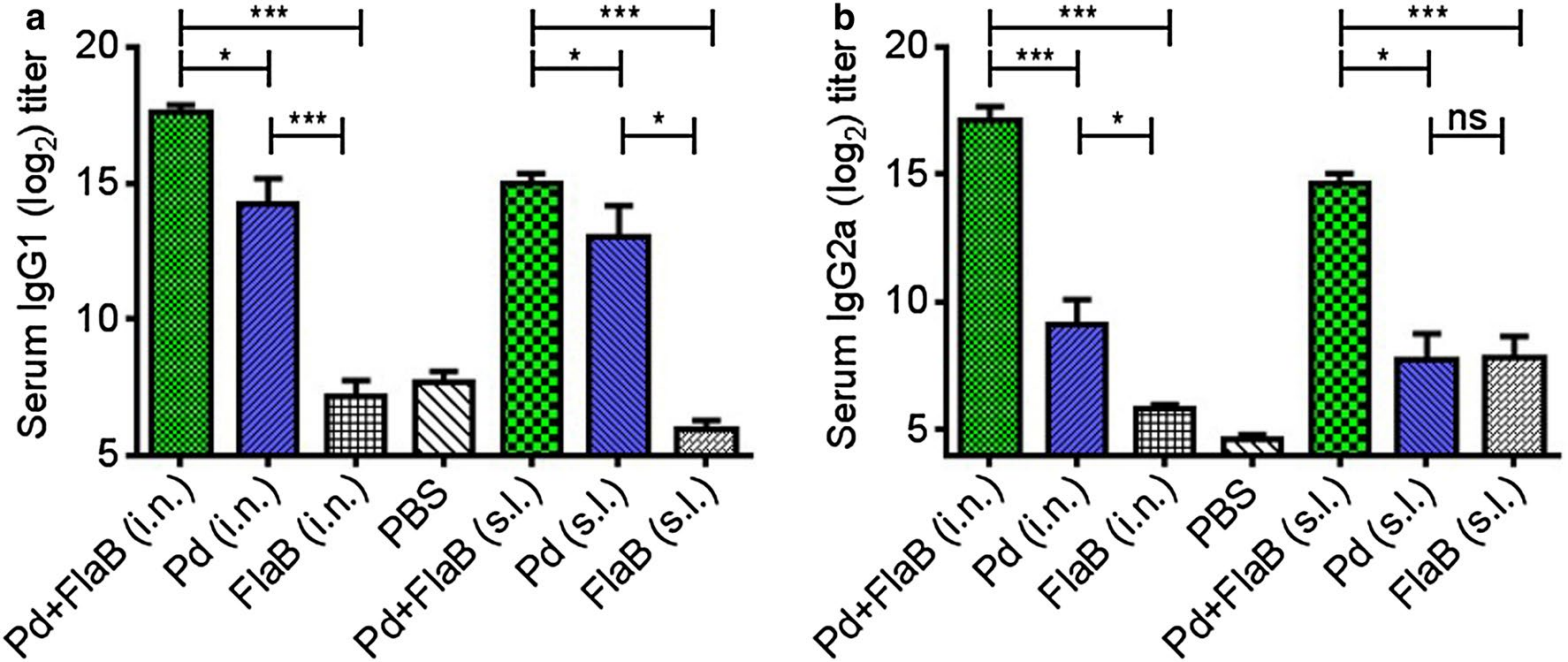
Induction of Th1/Th2 skewed immune responses post-vaccination. **a** Shows the serum-IgG1 levels while **b** shows the serum-IgG2a antibody levels 1 week after final vaccination of animals through i.n. or s.l. routes. Statistical differences were compared by the Student’s *t* test for unpaired means. *P < 0.05, **P < 0.01, ***P < 0.001, ^ns^not significant. The *error bars* represent the standard error of mean (SEM)

We further delineated the type and magnitude of immune response induced upon vaccination with various antigen combinations. Pd given through i.n. route induced a significant level of IgG1 (P < 0.001) (Fig. 3a) as well as IgG2a (P < 0.05) (Fig. 3b) antibody titers. On the other hand, Pd given through s.l. route induced significant titers of IgG1 (P = 0.005) (Fig. 3a) but not IgG2a (P = 0.475) (Fig. 3b). Evidently Pd alone induced T_H_2 skewed humoral immune response as the levels of IgG1 antibodies induced through i.n. or s.l. route were significantly higher than the titers of IgG2a induced through respective routes (P < 0.01). Incorporation of FlaB into vaccine formulation as a mixture (Pd + FlaB) significantly enhanced the respective antibody titers in both immunization routes (P < 0.01) (Fig. 3a, b). More importantly, Pd + FlaB mixture skewed the Pd induced humoral immune response from T_H_2 type to a mixed T_H_1/T_H_2 type as a higher increase in IgG2a antibody titers was observed after vaccination with Pd + FlaB (P = 0.0004) (Fig. 3b). These results indicate that the nature of immune responses to Pd has been changed by the action of mucosal adjuvant FlaB. These results also indicate that appropriate immune responses can be directed to specific immune compartments by altering the antigen formulation with specific adjuvant or route used for immunization.

### FlaB potentiates Pd-specific cell mediated immune responses in systemic and local immune compartments

**Fig. 4:**
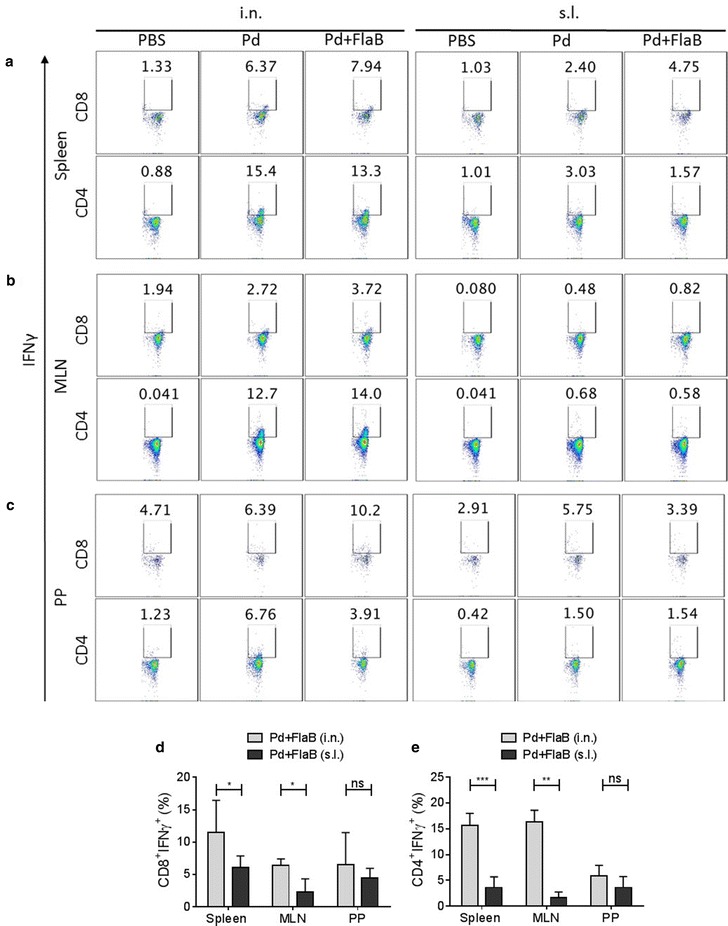
Induction of cell mediated immune responses in various immune compartments after immunization with Pd and Pd + FlaB through various routes. **a**–**c** Representative FACS micrograph showing percent of CD8^+^IFNg^+^ and CD4^+^IFNg^+^ cell numbers in mesenteric lymph nodes (MLN), payer’s patches (PP) and spleen after i.n. or s.l. immunization, as depicted in the picture. **d**, **e** The statistical analysis of cell percentages of IFNγ secreting CD8^+^ (**d**) and CD4^+^ (**e**) induced in spleen, MLN and PPs after Pd + FlaB immunization through i.n. and s.l. routes. Statistical differences were calculated by student’s *t* test for unpaired means. *P < 0.05, **P < 0.01, ***P < 0.001, ^ns^not significant. The *error bars* represent the standard error of mean (SEM)

Next we examined the cell mediated immunity generated in response to different antigen formulation and immunization routes. For this, 2 weeks after the third immunization, single cell suspensions from spleen, mesenteric lymph nodes (MLNs) and Peyer’s patches (PPs) were incubated with the purified Pd and Brefeldin A, after which cell activation and cytokine production were estimated by FACS analysis. Notably, while the percentages of IFNγ secreting lymphocytes were negligible in PBS group, a significant induction of CD8^+^IFNγ^+^ T lymphocytes was observed in spleen and MLNs after i.n. immunization with the Pd vaccine that was further significantly enhanced in Pd + FlaB group (Fig. 4a, c). Though the Pd vaccine by itself failed to induce a significant CD8^+^IFNγ^+^ T cell response in PPs, combination with FlaB resulted in enhanced response in PPs (Fig. 4b). On the contrary, in s.l. immunized animals, the levels of IFNγ induced in CD8^+^ cells after Pd or Pd + FlaB immunization remained insignificantly different from each other in all the three organs tested (Fig. 4a–c). Very interestingly, the levels of CD4^+^IFNγ^+^ T cells induced in all three organs after i.n. immunization with Pd or Pd + FlaB were not significantly different from each other though were higher than the PBS group (Fig. 4a–c). When compared across the immunization routes, in Pd + FlaB immunized animals, CD8^+^IFNγ^+^ (Fig. 4d) and CD4^+^IFNγ^+^ T (Fig. 4e) cell responses in spleen and MLN of i.n. immunized animals was significantly higher than those observed after s.l. immunization while such significant difference in the two T cell responses was not observed in PPs between i.n. and s.l. immunization routes. These data indicate that Pd vaccine given through i.n. route induces stronger CD8^+^IFNγ^+^ and CD4^+^IFNγ^+^ cell responses in spleen and MLN but not in PPs while combination with FlaB resulted in significant enhancement of respective responses (Fig. 4a–c). It is noteworthy that the CD8^+^ and CD4^+^ cell populations observed here may be constituted of other non-lymphocytic cells that may also be positive for these markers. Nonetheless, proportion of such cells in the tissues studied remain substantially low. Hence, these data clearly indicate that the i.n. route is better for the induction of IFNγ producing Pd-specific immune cells in the spleen and MLN than the s.l. route. But s.l. immunization appeared as efficient as the i.n. route in inducing IFNγ producing CD8^+^ and CD4^+^ cells in PPs.

### FlaB induces similar systemic immune responses compared to alum and CFA, while inducing significantly higher secretory antibody responses in feces

**Fig. 5:**
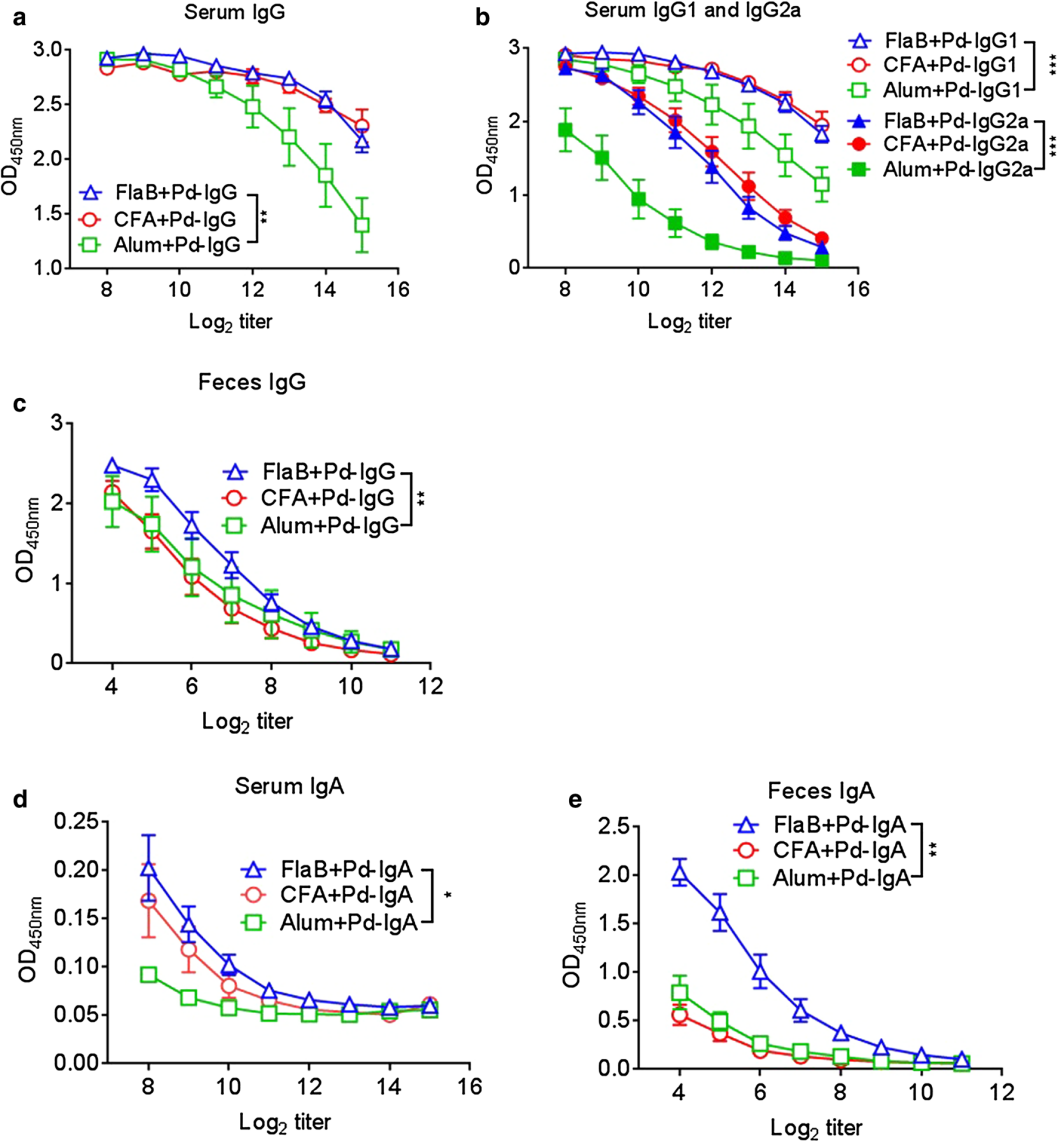
Comparison of various adjuvants for antibody induction. **a**, **b** The kinetics of appearance of IgG and IgG subclasses after immunization with Pd-vaccine mixed with various adjuvants and given through subcutaneous routes. **c** Comparative fecal IgG levels induced after final vaccination. **d**, **e** The respective serum and fecal IgA levels induced after vaccination with Pd combined with different adjuvants. *P* values were calculated by the paired Student’s *t* test. *P < 0.05, **P < 0.01, ***P < 0.001. The *error bars* represent the standard error of mean (SEM)

We compared the adjuvant effects of TLR5 agonist FlaB with already very well documented vaccine adjuvant alum and CFA. For objective comparison, same dose of antigen mixed with respective adjuvant was inoculated subcutaneously three times. One week after the final immunization, feces and serum samples were procured for antibody estimation by ELISA. We found that, compared to alum, FlaB and CFA induced significantly higher levels of IgG in serum (Fig. 5a) (P < 0.01). Upon further evaluation of subclasses of IgG antibodies induced by various adjuvants, we found that FlaB as well as CFA tended to induce high levels of mixed IgG1 and IgG2a antibody responses, whereas alum induced high levels of IgG1 (Fig. 5b). Interestingly, the levels of IgG induced by FlaB + Pd in feces were significantly higher than the other two adjuvants (Fig. 5c) (P < 0.01). It was interesting to note that subcutaneous immunization with FlaB + Pd induced high fecal IgG levels (Fig. 5c), while such antibodies were not detected upon giving FlaB + Pd through i.n. or s.l. routes (data not shown). Besides, we also found that FlaB induced high levels of IgA in serum that were significantly higher than those in CFA and alum groups (Fig. 5d) (P < 0.05). Moreover, the levels of fecal IgA induced by FlaB were significantly higher than that induced by alum and CFA (Fig. 5e) (P < 0.01). Hence, these results indicate that bacterial flagellin acts as a strong adjuvant that induces high levels of both secretory as well as virus binding antibodies in serum and feces even when administered systemically.

## Discussion

In the present study, we have shown that NoV specific P domain (Pd) recombinant vaccine, produced in *E. coli* system, effectively induced substantial humoral immunity in mice. The combination of Pd with the mucosal adjuvant FlaB [18] enhanced the humoral immune responses in a TLR5 dependent manner and also induced significant cell mediated immunity in systemic and local immune compartments. In general, the route of vaccination is critical for successful immunization outcomes against specific infections [21]. Most parenterally administered vaccines fail to induce local mucosal immune responses that act as a first line of defense against pathogens that invade through gastrointestinal surfaces. An important feature of present study is the information on the kinetics of antibody induction after vaccination through intranasal and sublingual routes. In agreement with previous reports, the immunization through intranasal as well as sublingual routes seems to induce considerable systemic as well as local antibody responses [22]. However, the Pd vaccine given through i.n. route induced higher IgG antibody titers after two immunizations, which were significantly higher than the antibody titers induced after s.l. immunization. On the other hand, secretory IgA antibody levels in feces were higher after the s.l. immunization compared with the i.n. route. The difference in vaccine efficacy given through two routes can be attributed to the initial tissue types associated with antigen capture. Nasal associated lymphoid tissue (NALT) is a secondary lymphoid organ with organized cell clusters while the sublingual mucosa is a non-organized type II mucosal tissue with dispersed immune cells that lack an organized cell clustering. However, combination of Pd with the mucosal adjuvant FlaB seems to overcome this hurdle and induce high antibody titers after two immunizations that might be due to the induction of immune responses mediated through the epithelial cells of buccal mucosa [23] or indirect activation of innate immune cells [24].

Direct effects of flagellin on B cell activation and antibody production [25] have been refuted variously [26], while stimulatory effects of flagellin on dendritic cells and T cells have been reported to promote a dramatic increase in T cell dependent antibody production [27]. The humoral responses enhanced by FlaB were characterized by high levels of IgG1 and IgG2a that are required to provide complete protection against the targeted pathogen [26]. Like many other subunit vaccines, the Pd vaccine administered through either mucosal route (i.n. or s.l.) induced a T_H_2 skewed immune response resulting in higher IgG1 over IgG2a titers. However, addition of FlaB to the formulation resulted in the induction of T_H_1 type immune response providing a mixed T_H_1/T_H_2 antibody response. We envisage that the multifunctional immune response generated after immune-potentiation by FlaB will provide superior efficacy in preventing NoV infections in human and animals. Moreover, we have previously shown that FlaB is a very effective and safe mucosal adjuvant as after i.n. immunization it does not accumulate in olfactory tissues and the central nervous system [18, 28].

In a few NoV vaccine studies done in humans [29] and chimpanzees [30] the correlates of protection indicate that both antibody and cell mediated immune responses are necessary to clear NoVs. In particular, in a mouse model, CD4 and CD8 T cells were required for clearance of virus from the intestine [31, 32]. Immune responses in mucosal tissues are governed by the nature of the antigen, the type of APCs involved, and the local microenvironment. With most types of non-adjuvanted peptide/protein antigens, the ‘default’ immune response seems to be the T_H_2 type response that may cause active suppression of systemic immunity [14]. However, antigens and adjuvants, including most PAMPs sensed by mucosal APCs as ‘danger signals’ (e.g. TLR ligands), favor the development of stronger and broader immune responses engaging both the humoral-secretory and cellular immunity effector arms. In the present study we found that P domain based vaccine, given alone, did not induce a substantial cell mediated immunity. However, as shown previously for other antigens [33], combination of Pd vaccine with flagellin resulted in induction of significantly higher IFNγ secreting CMI. Interestingly, i.n. immunization using Pd+FlaB tended to induce much higher levels of IFNγ secreting CD8^+^ and CD4^+^ lymphocytes than the same antigen combination given through s.l. route. Though there is a consensus regarding flagellin’s ability to induce CD4^+^ T cell-mediated immune responses, the TLR5-dependent CD8^+^ immune response is less defined. Data in the present study support previous reports that testified the significant activation of CD8^+^ lymphocytes through TLR5 signaling [34, 35]. However, there are studies that did not note the induction of cytotoxic T cell responses after immunization with flagellin adjuvant [36] highlighting the fact that the nature and characteristics of co-administered antigen, and route of immunization should determine the type and magnitude of the resulting immune response. Hence, every candidate antigen should be tested with adjuvant rather than predicting effects based upon published results.

Alum is the most common adjuvant used in approved prophylactic vaccines [37]. However, the propensity of alum based adjuvants to induce a T_H_2-skewed immune response and their inability to induce cell mediated immune responses [38], limits wider application against diseases where T_H_1 or cytotoxic T cell responses are critical for immune protection. Hence, we compared the adjuvant effects of FlaB with alum and CFA. CFA is an experimental adjuvant composed of oil-in-water emulsion incorporated with killed mycobacteria and is not recommended for human usage because of severe adverse inflammatory reactions at the site of inoculation [39]. Nonetheless, it is one of the strongest inducers of antibody and cell mediated immune responses. We found that FlaB induced high levels of serum IgG that were comparable to IgG levels induced by CFA but were significantly higher than that induced by alum. We further found that the levels of IgG2a induced after alum vaccination were significantly less than that induced by FlaB or CFA, though the levels of IgG1 were comparable among three groups. Moreover, induction of high levels of secretory IgA antibodies in feces of FlaB immunized animals but not in other two groups established the superiority of FlaB as an adjuvant given through mucosal (i.n. or s.l.) or non-parenteral routes. Taken together, these results indicate the importance of use of an appropriate adjuvant such as FlaB for induction of protective immune reactions in local settings. These results, in conjugation with our previously published reports, further support the superiority of FlaB as a mucosal adjuvant in inducing the antigen specific antibody as well as cell mediated immune reactions in the local mucosal settings.

## Conclusions

We show that NoV specific immune responses could be significantly fortified by the use of TLR5 agonist when given through mucosal route. The data in the present manuscript also conclusively demonstrate that for induction of relevant immune responses in appropriate immune compartments, route of immunization is a decisive factor. These results pave a way for the development of a relevant vaccine for human as well as veterinary use, in which NoV induced diarrhea and foodborne infections are a major cause of distress and financial loss.
